# Climate Change Threatens Micronutrient Density of European Winter Wheat

**DOI:** 10.1002/advs.202513322

**Published:** 2026-04-27

**Authors:** Da Cao, Jennifer Michel, Eline Lorer, Markus Weinmann, Jacques Le Gouis, Claire Léon, Sibille Perrochon, David Alvarez, Vincent Leemans, Iñaki Balanzategui Guijarro, Jordi Moya Laraño, Sara Sánchez Moreno, Symanczik Sarah, Waibel Matthias, Hervé Vanderschuren, Cécile Thonar, Pierre Delaplace, Dominique Van Der Straeten

**Affiliations:** ^1^ Laboratory of Functional Plant Biology Department of Biology Faculty of Sciences Ghent University Ghent Belgium; ^2^ Plant Genetics and Rhizospheric Processes Laboratory, Gembloux Agro‐Bio Tech, TERRA Teaching and Research Centre University of Liège Gembloux Belgium; ^3^ Forest & Nature Laboratory Department of Environment Ghent University Melle Belgium; ^4^ Institute of Crop Science, Nutritional Crop Physiology University of Hohenheim Stuttgart Germany; ^5^ UMR GDEC, INRAE Université Clermont Auvergne Clermont France; ^6^ Functional and Evolutionary Ecology Estación Experimental de Zonas Áridas (EEZA‐CSIC) Almería Spain; ^7^ Department of Environment and Agronomy National Institute for Agricultural and Food Research and Technology (INIA‐CSIC) Madrid Spain; ^8^ Department of Soil Sciences Research Institute of Organic Agriculture (FiBL) Frick Switzerland; ^9^ Tropical Crop Improvement Laboratory Department of Biosystems, KU Leuven Belgium; ^10^ Agroecology Laboratory Faculty of Sciences Université Libre de Bruxelles (ULB) Brussels Belgium

**Keywords:** B vitamins, climate change, micronutrient quality, minerals, winter wheat

## Abstract

Micronutrients are vital for human health. Wheat is a major staple crop and a significant source of minerals and B‐vitamins. The impact of multifactorial climate change on their content remains largely unknown, introducing uncertainty to human nutrition and well‐being. Here, we used an Ecotron to evaluate micronutrient levels in European winter wheat (*Triticum aestivum* var. Asory) under historical and projected climate conditions, incorporating gradients of atmospheric CO_2_, temperature, precipitation, and light intensity representative of ongoing climate change in Western Europe. Our findings indicate that future climates will strongly diminish multiple minerals and B‐vitamins in grains, thereby posing a significant challenge for global public health.

## Graphical abstract

Micronutrients are vital for human health. Wheat is a major staple crop and a significant source of minerals and B‐vitamins. The impact of climate change on their content remains largely unknown. We evaluated micronutrient levels in European winter wheat grown under historical and projected climate conditions. Our findings indicate that future climates will strongly diminish multiple minerals and B‐vitamins in grains, thereby posing a significant challenge for global public health.

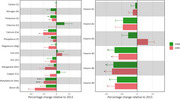

## 1 Introduction

Perhaps the most daunting challenge of the 21st century is to eradicate all forms of hunger (United Nations Sustainable Development Goal 2, SDG2) [1]. Presently, about one‐third of the world population faces micronutrient malnutrition (hidden hunger) resulting from vitamin and mineral deficiencies, while 720 million people are undernourished [1, 2]. Malnutrition remains a global challenge due to various factors, including the further growing world population and the recent COVID‐19 pandemic [1]. Unfortunately, this precarious situation is expected to be further exacerbated by global climate change. Modeling approaches project that climate change can reduce crop yields significantly in the next few decades [3, 4, 5]. In addition, the nutrient level of staple crop products is negatively affected by rising atmospheric CO_2_ concentrations—a major driver of climate change [6, 7, 8]. These crops, such as wheat and rice, are losing macronutrients, including protein, as well as essential minerals, like iron (Fe) and zinc (Zn). A free air CO_2_ enrichment (FACE) study in rice also demonstrated a consistent decline between 20%–40% in several B‐vitamins (B1, B2, B5, B9) [8]. The latter poses a serious health risk since humans fully rely on their daily diet for these micronutrients. Micronutrient deficiencies are associated with impaired physiological function, cognitive decline, various chronic diseases, and mortality, and currently affect more than 2 billion people worldwide [9, 10, 11, 12, 13].

However, these results, while valuable, may not fully capture the intricate and synergistic effects of climate change on agricultural systems in real‐world conditions. Climate change presents a complex set of environmental challenges, not only including elevated CO_2_ levels, but also temperature fluctuations, changes in precipitation and humidity, extreme weather events, as well as soil quality variations due to soil erosion and changes in the microbial communities of the rhizosphere [14, 15]. In particular, the combined effects of several environmental factors on crop nutrient levels warrant thorough investigation, even though integration of all these variables in an empirical setting is challenging [16].

Recent advances in analytical techniques enable the simultaneous quantification of a wide range of grain micronutritional traits, including B vitamins and mineral elements, alongside plant phenotypic responses. These developments have substantially reduced measurement time and facilitated comprehensive profiling of micronutrient levels. For example, validated methods for the simultaneous quantification of water‐soluble vitamins (primarily B vitamins) and fat‐soluble vitamins have been established across diverse food matrices [17, 18, 19, 20]. Simultaneous profiling of mineral contents is also feasible across various food matrices [21, 22]. Together, these advances provide new opportunities to comprehensively assess crop nutritional quality under complex environmental conditions.

Despite these technological improvements, a critical knowledge gap remains in understanding how multiple interacting environmental factors jointly influence micronutrient levels in crops. Decades of FACE experiments have provided important insights into the effects of CO_2_ enrichment on future crop productivity [23]. However, in such field studies, control over environmental factors is limited. Galani et al. [24] attempted to simulate climate change conditions—temperature, CO_2_, and drought‐ within a controlled growth chamber. They found that these combined abiotic stresses led to increased concentrations of protein, Fe, and Zn in wheat seeds. Yet, the environmental settings were kept constant daily, omitting the natural diurnal and seasonal variability present in natural ecosystems.

Compared with FACE experiments and standard controlled growth chambers, an Ecotron is a state‐of‐the‐art controlled environment facility that allows to realistically implement multifactorial climate change scenarios, including fluctuations in diurnal and seasonal sunlight intensity and spectral composition, as well as alterations in temperature and humidity [25]. The TERRA Ecotron used in this investigation offers an exceptional platform to study the effects of climate change with high temporal resolution as well as experimental flexibility, with key environmental parameters adjustable every few minutes [25, 26]. Moreover, an Ecotron can not only replicate the past and present climate conditions but also simulate predicted future scenarios, hence providing an excellent tool for climate change studies.

This work aims to uncover the effects of future climate conditions on grain micronutrient concentrations in European winter wheat using the Ecotron facility. Three meteorological conditions were applied to represent a climate‐change gradient for Western Europe, spanning from historical conditions (2013) to projected future scenarios (2068 and 2085), characterized by a continuous increase in the hydrothermal index [26]. Thirteen mineral elements and six B vitamins were successfully quantified in harvested wheat grains, and these data were integrated with phenotypic traits to examine associations between plant growth responses and grain micronutrient changes.

## 2 Results

**FIGURE 1:**
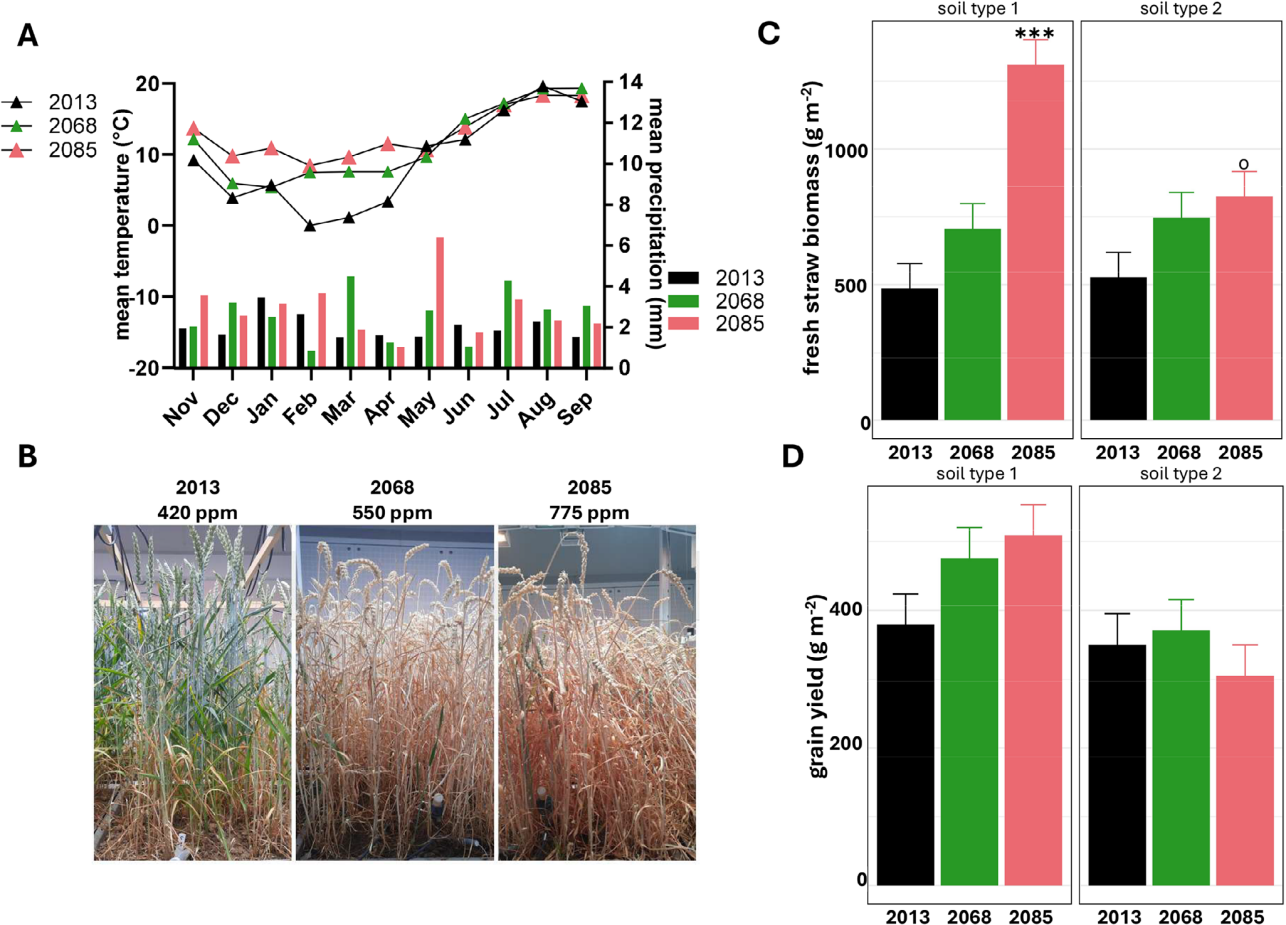
Projected meteorological conditions used in the Ecotron and their impact on wheat phenology. (A) Projected meteorological conditions including monthly mean precipitation and temperature for 2013, 2068, and 2085. (B) CO_2_ levels and corresponding phenotypes of winter wheat (Asory) grown under three meteorological conditions, 249 days after sowing. Total fresh weight of straw (g m^−2^) (C) and grain yield (g m^−2^ at 14% grain humidity) (D) of winter wheat plants harvested under different meteorological conditions on two soil types. Values are mean± SE, n = 4. Asterisks indicate significant differences in comparisons between future conditions and the year 2013 as a control (^*^ *p* < 0.05; ^**^ *p* < 0.01; ^***^ *p* < 0.001, linear mixed model). Circles indicate marginally significant differences, with adjusted *p* values ranging from 0.1 to 0.05. Specifically, for straw fresh weight in 2085: *p* = 0.00001 for soil type 1 and *p* = 0.07 for soil type 2.

As a representative of winter wheat in Europe, the wheat variety Asory was grown in the TERRA‐Ecotron facility (Figure S1). Asory has been chosen based on its wide use in Europe, from Northwestern, Western, to Central Europe, related to its broad adaptability, including drought resilience. Previous field trial results show its robust performance under current and future climate conditions [27, 28]. The climate change gradient was established using historical data from Belgium for 2013 and predicted future meteorological conditions for 2068 and 2085 using the ALARO‐0 model and the RPC8.5 scenario [26, 29]. The simultaneously controlled main environmental factors comprised atmospheric CO_2_ (constantly at 420, 550, and 775 ppm), temperature (adjusted every 5 min; annual mean: 7.6, 10.2, and 12.1(C), precipitation level (adjusted daily; annual mean: 2.1, 2.4 and 3.0 mm·day^−1^), and light intensity (adjusted every 5 min; average irradiation: 157, 163.6 and 148.9 µmol m^−2^ s^−1^)(Figure 1A). The experiment also used two soil types of contrasting soil organic matter content, with soil type 2 containing higher organic matter than soil type 1 [26].

Details of plant growth performance were extensively described in our previous publication [26]. Briefly, wheat plants grown under future meteorological conditions exhibited shorter life cycles consistent with observations in rice under global warming and elevated CO_2_ [30]. In addition, wheat plants showed increased fresh straw biomass and grain yield in both soil types compared with those grown under conditions in 2013, except for grain yield in soil type 2, which remained unchanged (Figure 1C, D). Interestingly, soil type showed a strong effect on both grain yield and straw biomass, with a more pronounced increase under future climate scenarios in soil type 1 compared with soil type 2 (Figure 1D).

**FIGURE 2:**
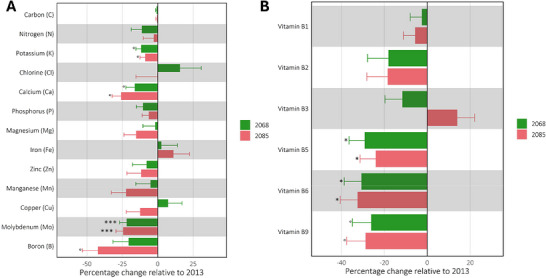
Relative changes of carbon, nitrogen, mineral, and B vitamin concentrations in wheat grains harvested under projected future meteorological conditions. Relative carbon, nitrogen, and mineral (A) and B vitamin (B) concentration changes in wheat grains harvested under future meteorological conditions (2068 and 2085) compared to 2013. Values are mean± SE, *n* = 16. Asterisks indicate significant differences in comparisons between future conditions and the year 2013 as a control (^*^ *p* < 0.05; ^**^ *p* < 0.01; ^***^ *p* < 0.001, linear mixed model). Specifically, *p* = 0.00008 and 0.00003 for Mo in 2068 and 2085, respectively; *p* = 0.047 for vitamin B5 and 0.032 for vitamin B6 in both 2068 and 2085; *p* = 0.047 for vitamin B9 in 2085. Circles indicate marginally significant differences, with adjusted *p* values ranging from 0.1 to 0.05. Specifically, *p* = 0.097 for K in both 2068 and 2085; *p* = 0.083 and 0.053 for Ca in 2068 and 2085, respectively; *p* = 0.062 for B in 2085; and *p* = 0.059 for vitamin B9 in both 2068 and 2085.

To evaluate the nutritional value of wheat grains harvested under future meteorological conditions, we measured the concentration of 17 elements and 7 essential B vitamins, all critical to human health. Strikingly, we observed an overall concentration decline for most micronutrients in grains harvested under future meteorological conditions compared to the conditions in 2013 (Figure 2; Figure S2, Tables S1 and S2). It has been reported that elevated CO_2_ negatively affects the mineral content, particularly nitrogen (N)—which is tightly linked to protein levels—not only in crops but also in many other plant species [6, 7, 8, 31]. Here, we found no significant decreases of N concentration in wheat grains under future meteorological conditions compared to 2013 (Figure 2A and Figure S2A). However, the concentrations of most measured minerals showed a clear trend of decrease compared to 2013, except for chloride (Cl), Fe, and copper (Cu) (Figure 2A). The level of molybdenum (Mo) declined significantly (22% to 24%) in wheat grains harvested under both future conditions compared to 2013. While elevated CO_2_ has been reported to significantly reduce N and Fe levels in wheat [6], we did not observe this effect in our experiments. This may be due to the different soil type, plant life cycle, and/or wheat variety used here, but it is also possible that other climate factors, such as precipitation or rising temperature, positively regulate N and Fe accumulation in wheat seeds, offsetting the reductions caused by elevated CO_2_ as observed previously [32, 33, 34].

Strikingly, all B vitamins consistently declined in the two future climate scenarios compared to 2013, except for vitamin B3 (niacin) in 2085 (Figure 2B and Figure S2B). Vitamins B5 (pantothenic acid) and B6 (pyridoxine) showed significant decreases ranging from 25% to 30% under future meteorological scenarios. Interestingly, these decreasing trends remained consistent across the two soil types for B vitamins and minerals, suggesting that climate change‐induced declines in nutritional content occur across soils with different characteristics (Figure S2), calling for detailed studies to investigate the climate change mitigation potential of soil management [26].

In addition, future climate conditions were found to alter protein fractions essential for dough rheology and processing quality. Previous studies have shown that elevated CO_2_ and heat stress reduce gliadin levels and the gliadin/glutenin ratio in wheat [32, 35]. Our findings align with these reports, showing overall reductions under projected climate scenarios (Figure S3).

To investigate potential mechanisms associated with grain nutrient decline, correlation analyses were performed between grain micronutrient concentrations and a range of plant physiological and molecular characteristics, including straw biomass, grain yield, thousand kernel weight (TKW), grain size parameters (area, length, and width), foliar mineral content, plant height, leaf area, and photosynthetic efficiency (Figure S4).

A growth‐associated dilution effect was observed for several types of micronutrients in wheat grain. For minerals, grain width and TKW were significantly negatively correlated with grain Ca and K concentrations, while grain area and straw biomass were also negatively correlated with grain Ca concentration. In addition, plant height and leaf area showed significant negative correlations with grain Mn, B, and Mo concentrations (Figure S4). In contrast, no significant correlations were detected between mineral concentrations between leaves and grains (Figure S4). Interestingly, grain yield was positively correlated with grain Mg and Mo concentrations, whereas grain length was positively correlated with grain Fe (Figure S4).

Among B vitamins, vitamins B5 and B6 showed the most pronounced declines in future climate scenarios (Figure 2B) and exhibited strong and significant negative correlations with grain area, width, TKW, straw biomass, and leaf area (Figure S4). In addition, vitamin B6 showed significant negative correlations with plant height, leaf area, and photosynthesis efficiency, further supporting a growth‐associated dilution effect. Finally, low‐molecular‐weight glutenins were positively correlated with plant height, resulting in a negative correlation between the gliadin‐to‐glutenin ratio and plant height (Figure S4).

## 3 Discussion

Our results suggest that future climate change may exacerbate the prevalence of hidden hunger, even without extreme climate events such as heavy flooding and drought, threats to agricultural yields that are becoming increasingly frequent [36, 37]. The simulated future meteorological conditions exert strong negative effects on both B‐vitamin and most mineral concentrations in wheat grains. This could pose a significant nutritional concern, particularly in population groups where hidden hunger is severe, and staple crops like wheat and rice are primary sources of these nutrients. In this context, micronutrient levels in staple crops are already far below the human Reference Daily Intake (RDA), and climate change could exacerbate these deficiencies [37]. A growth‐associated dilution effect was observed for several micronutrients. Grain size, plant height, and leaf area were significantly negatively correlated with several minerals (Ca, K, Mn, B, and Mo) and B vitamins (B5 and B6). The latter may affect plant resilience to several abiotic stresses [37, 38, 39, 40, 41]. Notably, the magnitude of these declines varies among individual nutrients and vitamins, reflecting possible differences in biosynthesis, uptake, (re)mobilization capacity, and biochemical regulation during grain filling. Climate change may therefore differentially affect specific nutrients from a mechanistic viewpoint, even when overall dilution patterns have been observed in the current study (Figure 2). Importantly, while the studied variety Asory is representative of current European winter wheat genotypes, it is possible that other varieties may respond differently, highlighting the need for future screening efforts across a broader genetic population [6, 8, 30, 42].

The effect of elevated CO_2_ on macronutrients and minerals has been studied previously. An increase in crop biomass and yield through enhanced carbohydrate accumulation was observed, while reducing nutrient concentrations, especially for nitrogen and protein, a phenomenon referred to as the CO_2_ dilution effect [7, 43]. Consistent declines in N and protein content have been reported across FACE experiments [7, 44], whereas responses of other macro‐ and micronutrients remained variable [6, 8, 45, 46]. Lieffering et al. [45] observed no significant changes in 12 minerals in rice under elevated CO_2_, except for a consistent decrease in N across two years of FACE field trials. In contrast, Myers et al. [6] and Zhu et al. [8] reported significant reductions in N, Fe, and Zn concentrations under elevated CO_2_ in multiple crop species. In a three‐year field study on sorghum and soybean, elevated CO_2_ consistently reduced N concentrations in two of the three years, while responses of Mg, K, Fe, and Ca varied by year and by species but mainly declined, while other minerals (Zn, Cu, and Mn) remained unaffected [46]. In comparison, the effects of elevated CO_2_ on vitamins were poorly studied. A FACE study reported reductions in vitamin B1, B2, B5, and B9 in 9 different cultivars of rice, while B6 remained unaffected [8].

The current study does not only considers the effects of elevated CO_2_ but also encompasses several other environmental factors that undergo alterations as a result of climate change, including temperature and precipitation, using an Ecotron facility [26]. The nutrient reductions observed here cannot be explained solely by a CO_2_‐specific dilution effect, even though a growth‐associated dilution effect was observed (Figure S4). The grain N concentration decreased strongly in 2068 compared with 2013, but not in 2085, which has a higher simulated atmospheric CO_2_ concentration compared with 2013 (Figure 2). Furthermore, the decline in grain N concentration showed no significant association with any plant growth or yield traits (Figure S4), in contrast to patterns commonly reported in FACE experiments [7]. Moreover, consistent decreases in micronutrient concentrations were observed, irrespective from soil type, as both soils showed similar negative effects (Figures S2 and S3). In contrast, biomass and yield responses to future climate scenarios differed between soil types (Figure 1C, D). These observations suggest that future climate change may reduce seed nutrient levels through multiple mechanisms, in addition to the CO_2_ dilution effect, such as diminished root uptake, impaired nutrient assimilation, reduced transpiration, and a shortened growth phase. In addition to elevated CO_2_, other climate parameters, particularly rising temperature and altered precipitation patterns, are known to modulate these physiological processes and thereby influence grain nutrient accumulation [32, 33, 34]. Specifically, rising temperature can directly affect root mineral uptake, phloem transport, and grain‐filling dynamics, leading to altered nutrient allocation to grains [47, 48, 49]. Likewise, changes in precipitation pattern could induce moisture or drought stress, which strongly impact root architecture, nutrient assimilation, and ultimately affect crop growth and grain nutrient concentration [34, 50]. However, since changes in root nutrient uptake, transpiration‐driven mass flow, phloem loading/unloading, and remobilization during grain filling were not directly measured in this study, our correlation analysis between grain micronutrient concentrations and plant physiological and molecular characteristics remains indicative of physiological processes potentially underlying the observed nutrient declines rather than establishing a causal relationship. Future research incorporating isotopic tracing, nutrient uptake rates, and targeted measurements of transport proteins and enzyme activities would be required to elucidate the precise mechanisms. Moreover, while the future climate scenarios of this experiment incorporated a gradient in mean temperature, mean precipitation, and hydrothermal index to reflect increasing overall meteorological stress, short‐duration extreme events such as heatwaves or drought episodes during sensitive growth periods like grain filling may exert disproportionate impacts on grain micronutrient composition that may not be fully captured by seasonal indices. Since these events are expected to become more frequent under future climate change [51, 52], future studies employing high‐temporal‐resolution climate forcing or targeted stress experiments during critical growth stages would be valuable to better quantify their specific contributions to micronutrient variability.

Importantly, the micronutrients studied, both minerals and vitamins, are essential not only for human nutrition but also for the regulation of plant development and stress resilience. Minerals serve as structural components of cells, constituents of organic molecules, and cofactors for numerous enzymes and transcription factors, playing key roles in photosynthesis, nutrient assimilation, and stress responses [51, 52]. B vitamins function as critical coenzymes in central metabolic pathways, including carbon and nitrogen metabolism, and support plant growth, development, and resilience to environmental stresses [38, 53, 54]. Studying changes in these nutrients is therefore crucial for both nutritional quality and crop performance under changing climate conditions. Further research is needed to elucidate the molecular mechanisms underlying climate‐induced declines in micronutrient content.

The United Nations aims to achieve zero hunger, eliminate food insecurity, and address all forms of malnutrition by 2030 (https://www.un.org/sustainabledevelopment/hunger/). However, this is an immense challenge. Our findings indicate that this precarious situation could be further exacerbated by climate change. Given the urgent need for an adequate nutritional supply, we argue that governments and stakeholders, including crop bioengineers, breeders, and farmers, should focus not only on food production (yield) but also on food quality (nutrient content) to combat hidden hunger and ensure nutritional security [55]. Prioritizing crop biofortification and adopting innovative farming practices are essential for developing nutrient‐rich crop varieties as part of sustainable cropping systems that are resilient to climate change.

## 4 Experimental Section

### 4.1 Ecotron Setup

The Ecotron setup has been detailed previously [26]. The TERRA‐Ecotron setup represents one of the indoor facilities closest to real agricultural field conditions. Important limitations of indoor systems are largely mitigated in this experimental setup. The TERRA‐Ecotron has six controlled environment rooms (CERs), in each of which nine cubes with soil monoliths of 125 L each (50×50×50 cm) were placed. Hence, we sampled 54 intact soil monoliths from two fields, 125L each. This approach allowed to closely represent field conditions, both from the point of view of soil heterogeneity and soil microbiome. Furthermore, a soil depth of 50 cm allows to capture of realistic root development. A global synthesis of root distributions across terrestrial biomes reports that ∼75% of root biomass is typically found in the top 40 cm, with cereals often showing even higher concentrations in the upper layers due to nutrient availability near the surface [56]. Similarly, multiple field studies on winter wheat confirm that the bulk of root length distribution is in the top 30 cm, while only a few roots extend much deeper [57]. We used a sowing density (308 seeds m^−2^) similar to field conditions, thereby realistically reproducing interspecies competition. Cubes were weeded manually to reduce plant‐weed competition, as a farmer would in the field. Herbicides were not used, nor any other chemical plant protection agent, so as not to disturb biological mechanisms and leave the soil microbiome intact.

The experiment was implemented in 2022/23. Winter wheat (Triticum aestivum L. var. Asory) was grown under three different meteorological conditions (2013, 2068, 2085), each reproduced in two CERs. This was crossed with the factor “soil” at two levels (low organic matter in soil type 1 and high organic matter in soil type 2). Under each climate scenario, the wheat was grown in two different soil types extracted from two agricultural fields in the Walloon region in the South of Belgium, which were both classified as Aba(b)0 in the Belgian soil classification system. Soil type 2 had received 2x more organic matter than soil type 1 in the years prior to soil excavation, which resulted in significantly enhanced soil organic carbon and nutrient contents, while soil type 1 had higher sand content [26]. This experimental setup resulted in a total of six modalities (2013.soil type 1, 2013. soil type 2; 2068. soil type 1, 2068. soil type 2; 2085. soil type 1, 2085. soil type 2), each presented with eight replicates. Amongst these eight replicates per modality, soil from four cubes was sampled at two plant growth stages (tillering and elongation) for analysis of soil meso‐fauna, and two additional cubes were sampled at the same time points for root analysis with small soil augers (d = 5 cm, h = 20 cm), while the remaining two cubes were kept intact and undisturbed until the final harvest. For analysis of plant biomass production, including grain yield, the two latter two categories are considered as they represent the most unbiased and undisturbed plant growth conditions. Grain quality analysis (micronutrients, vitamins, and protein composition) was performed on all eight replicates per modality.

### 4.2 Climate Scenarios

The detailed climate scenarios represent the climate change gradient predicted for Western Europe during the current century. To identify representative years, historical data of continuous climate observations from the Ernage meteorological station (Gembloux, Belgium, since 1980) were evaluated, and future meteorological conditions were predicted under the RCP8.5 scenario using the ALARO‐0 model for the two time windows 2040–2070 and 2070–2100 [29]. Three years were chosen along a continuous gradient of increasing temperature (7.6‐10.2‐12.1°C), precipitation (2.1–2.4–3.0 mm), hydrothermal index (4.0–4.5–4.7), and atmospheric CO_2_‐concentrations (420‐550‐775 ppm). A global temperature rise of 5°C over 70 years is within the upper range of IPCC projections under high‐emission scenarios. We use an RCP8.5 scenario, which is based on continued heavy reliance on fossil fuels without mitigation. According to IPCC AR6, under SSP5‐8.5 (‘very high’ emissions), global mean surface temperature could increase by 3.3–5.7°C by 2081–2100 relative to preindustrial times (1850–1900) [58]. The specific years 2013, 2068, and 2085 were chosen as representative conditions along the climate‐change gradient; they do not imply that these exact meteorological sequences will occur in those calendar years, but rather that they embody the projected mean trends and variability for the respective periods under the RCP8.5 scenario [26].

The ALARO‐0 model is a well‐established hydrostatic regional climate model (RCM) developed by the Royal Meteorological Institute (RMI) of Belgium. It is based on the ALADIN numerical weather prediction system [59]. It is designed for high‐resolution simulations, particularly over Europe, incorporating advanced parametrizations for processes like convection and precipitation to improve accuracy in extreme weather events [59, 60]. To materialize the future meteorological conditions in the TERRA‐Ecotron, the data of the ALARO‐0 model, which was provided by the RMI with 3‐h time steps (i.e., eight measures per day), was interpolated linearly to the 5‐min time steps required by the Ecotron controllers.

In relation to IPCC predictions and models, ALARO‐0 serves as a downscaling tool within the Coordinated Regional Climate Downscaling Experiment (CORDEX) framework, in support of IPCC‐AR6, including its European branch (EURO‐CORDEX). It refines coarser global climate model outputs to provide higher‐resolution regional projections for variables like temperature, precipitation, and extremes [29]. In this sense, ALARO‐0 is part of the IPCC as these downscaled simulations contribute to the ensemble of data used in IPCC assessments, enhancing the regional detail in reports that rely mainly on global Coupled Model Intercomparison Project (CMIP) ensembles for broader predictions. Therefore, we consider that predictions from the ALARO‐0 model can reflect plausible real‐world future scenarios, incorporating uncertainties like emission pathways, model biases, and natural variability. As a regional climate model validated within frameworks like EURO‐CORDEX, ALARO‐0 has demonstrated strong performance in simulating historical European climate patterns, including temperature, precipitation, and extremes, with results often aligning well with observations and outperforming some peers in aspects like extreme precipitation [29, 59, 60]. While the three representative years selected here under this framework provide a coherent high‐emission climate‐change gradient for Western Europe, future studies would benefit from multi‐model ensemble approaches such as those available in CMIP6/EURO‐CORDEX, which offer higher spatial resolution, more updated scenarios, and more detailed physical representations. Such comparisons would help quantify the effect of inter‐annual variability on wheat growth, nutrient uptake, and grain quality and reduce scenario uncertainty.

### 4.3 Plant Growth and Harvest

Wheat seeds were planted at a density of 308 seeds per square meter in the Ecotron facility, and various growth parameters, including plant height, leaf area, and chlorophyll fluorescence, were closely monitored throughout plant growth [26]. At BBCH30 and BBCH70, the youngest fully developed leaves of 3 plants per cube were collected in the four cubes designated to destructive harvest, and immediately frozen in liquid nitrogen for analysis of mineral contents. Plants and grains were harvested at full maturity (BBCH89), and after weighing, subsets of grains were immediately frozen in liquid nitrogen for nutrient analyses.

### 4.4 Elemental Analysis

Foliar and grain contents of C and N were determined on dried and milled samples via Elemental‐Analyzer (Elementar Vario EL, Elementar Analysensysteme GmbH, Langenselbold, Germany; VDLUFA‐Methodenbuch Band I, A 2.2.5: Bestimmung von Gesamt‐Stickstoff nach trockener Verbrennung (Elementaranalyse)). For further mineral nutrient analyses in wheat leaves and grains, dried and milled samples (ca. 200 mg dry matter) were mineralized by microwave (UltraClave V Fa. MLS Leutkirch, Germany) digestion with HNO3 [61]. In this sample solution, Ca, Cu, Fe, K, Mg, Mn, Na, P and Zn concentrations were measured by Inductively Coupled Plasma Optical Emission spectroscopy (ICP‐OES; Agilent 5110, Agilent Technologies, Santa Clara, California, USA)(VDLUFA‐Methodenbuch Band VII, Methode 2.2.2.6: Bestimmung von ausgewählten Elementen in pflanzlichem Material und Futtermitteln mit ICP‐OES). B, Co, Mo, Ni, and Se were measured by Inductively coupled plasma mass spectrometry (ICP‐MS; Perkin Elmer NexION 300 X, PerkinElmer, Waltham, MA, USA). Cl was extracted from the ground sample with hot water and measured by ion chromatography (IC; Integrion, Thermo Fisher Scientific, Waltham, MA, USA; VDLUFA‐Methodenbuch 2.2‐4.7.2.2 Ionenchromatographische Bestimmung von Chlorid). All analyses were conducted at the Core Facility University of Hohenheim, Analytical Chemistry Unit, Germany. Na and Co were in all samples below the detection limit at 20 and 0.025 mg kg^−1^, respectively. Partial values of Se and Ni were below the detection limit at 0.05 and 0.025 mg kg^−1^, respectively.

### 4.5 B Vitamin Analysis

An established protocol was followed for B vitamin analysis [17]. Briefly, approximately 50 mg of homogenized wheat grain powder, prepared from 10 g of ground seeds, were accurately weighed and extracted with 50 mm phosphate buffer containing corresponding internal standards for each group of B vitamins [17]. The extracts were incubated with protease for 1 h at 37°C and deactivated at 95°C for 10 min. After cooling down, acid phosphatase, β‐glucosidase, and rat serum were added and incubated for 2 h at 37°C to release related B vitamin derivatives. After deactivation at 95°C for 10 min, the extracts were filtered and transferred to amber vials for UHPLC‐MS/MS analysis. The biotin level was below the detection limit, so the results were not reported.

### 4.6 Protein Composition Analysis

Protein composition was determined after sequential extraction. Briefly, 66.6 mg of complete flour was mixed with 1 mL of 50 mM phosphate buffer (pH 7.8) containing 100 mM NaCl (30 min, 800 rpm, 4°C). To extract gliadins, the pellet was mixed with 1 mL 70% ethanol and centrifuged (10 min, 18,000 g, 4°C). This operation was repeated twice. After each centrifugation, the supernatant containing gliadins was collected and pooled. To extract glutenins, the resulting pellet was mixed with 0.5 mL of 25 mM borate buffer (pH 9.8) containing 50% propanol‐2 and 1% DTT (30 min, 1200 rpm, 50°C) and centrifuged (10 min, 18,000 g, 18°C). This operation was repeated once. After each centrifugation, the supernatant containing glutenins was collected and pooled. For stability, the glutenin fraction was alkylated with 4.6% of 4‐vinylpyridine (15 min, 60°C). The two glutenin subunits (High‐Molecular Weight Glutenin Subunit, HMW‐GS; Low‐Molecular Weight Glutenin Subunit, LMW‐GS) and four gliadin classes (α/β‐, γ‐, ω1,2‐, ω5‐gliadin) were quantified by reverse phase high performance liquid chromatography (RP‐HPLC) using an Agilent 1290 Infinity LC system (Agilent Technologies, California, USA) as described before [62]. Briefly, gliadin and glutenin extracts were filtered through regenerated cellulose syringe filters (0.45‐µm pore diameter, UptiDisc, Interchim, Montluçon, France), then 4 µl of each protein extract was injected into a C8 reversed‐phase ZORBAX 300 Stable Bound column (2.1 × 100 mm, 3.5 µm, 300 Å; Agilent Technologies) maintained at 50°C. Proteins were separated at a flow rate of 1 mL/min using linear solvent gradients from 24% to 50% acetonitrile containing 0.1% (v/v) trifluoroacetic acid over 13 min for gliadins, and from 23% to 42% over 25 min for glutenins. Proteins were detected by UV absorbance at 214 nm. Chromatograms were processed with ChemStation 10.1 software (Agilent Technologies), and the HPLC peaks corresponding to each of the four gliadin classes and the two glutenin subunits were identified following Wieser et al. [63]. All quantities were corrected for the extraction yield, which was estimated to be 93% for gliadins and 65% for glutenins extracted without SDS. Protein quantities were determined with a calibration curve based on the Dumas combustion method using a FlashSmart N Analyzer (ThermoScientific, Villebon‐sur‐Yvette, France).

### 4.7 Statistical Analysis

For grain nutrient analysis, the data for both soil types per climate were combined (n = 16 per climate, n_total_ = 48) to obtain the most representative subsample of the winter wheat population in this experiment. Growth parameters were measured in disturbed cubes only; hence, the yield and straw biomass analysis, and correlation analysis, used n = 4 replicates per modality (n_tot_ = 24). All data analyses were performed in R v.4.3.2 [64]. No data transformation was applied. Using the ‘lme4’ [65], we fitted linear mixed‐effects models to test the effects of future meteorological conditions (year – 3 levels: 2013 as a control, 2068, and 2085) on each quantified nutrient separately. We applied a consistent random effects structure with one random effect for growth chamber (6 levels), resulting in the following model structure:

Plant response ∼ Year + (1│chamber).

To compare model estimates of 2068 and 2085 to the control, we used ‘emmeans’ [66] to perform post‐hoc tests with false discovery rate (FDR) p‐value adjustment and Satterthwaite approximations for degrees of freedom [67]. To calculate the percentage change of a given response in 2068 or 2085 relative to 2013, we divided the respective model estimates (±SE) for these levels (i.e., the estimated mean change in 2068 or 2085 relative to the control 2013) by the estimate for the intercept (i.e., the estimated mean of the response in 2013).

In a second step, we studied the effects of future meteorological conditions in the different soils with the same random effects structure (see Figure S2, Tables S1 and S2):

Plant response ∼ Year*Soil + (1│chamber).

Post‐hoc tests were performed as described above for the models with year, soil, and year*soil interaction as main effects.

The raw data and diagnostic plots (Q‐Q, residual vs. fitted) are provided in the Supporting Information.

To evaluate potential links between plant growth and grain nutritional concentrations, a correlation analysis was performed using the corrplot package in R [68]. As some parameters violated normality assumptions, Spearman correlations were used, and p‐values were adjusted using FDR [67]. Only correlations with an adjusted p<0.05 are shown. The raw data are provided in the Supporting Information.

## Author Contributions

All authors were involved in experimental design and data interpretation. J.M. and V.L. performed the Ecotron experiment. D.C., M.W. and J.L.G. performed nutrient analyses. D.C., J.M. and E.L. analyzed the data. D.C. and D.V.D.S. wrote and coordinated the manuscript, including contributions from M.W. on mineral content, J.L.G. and J.M. on protein content, and E.L. and J.M. on statistical analyses. All authors critically reviewed the manuscript.

## Funding

DVDS is grateful to the Francqui Foundation for awarding her a Collen‐Francqui Research Professorship (STI.DIV.2022.0014.01). DVDS also acknowledges the Ghent University Special Research Fund (Bijzonder Onderzoeksfonds BOF) for financial support (BOF18‐GOA‐042). This research is part of the BIOFAIR project (www.biofair.uliege.be) funded through the 2019–2020 BiodivERsA joint call for research proposals under the BiodivClim ERA‐Net COFUND programme, with funding from the following organizations: Fonds Wetenschappelijk Onderzoek (FWO, Flanders, Belgium; FWO ERA‐NET G0H7320N), Fonds de la Recherche Scientifique (FNRS, Wallonia, Belgium; R.8001.20), Agence Nationale de la Recherche (ANR, France; ANR‐20‐EBI5‐0002), Agencia Estatal de Investigación (AEI, Spain; PCI2020‐120713‐2), Deutsches Zentrum für Luft‐ und Raumfahrt Projektträger (DLR‐PT, Germany; 16LC2028A), and Schweizerischer Nationalfonds (FNS, Switzerland; 31BD30_193869). EL ackowledges the Flanders Research Foundation (FWO) for funding (G078921N).

## Conflicts of Interest

The authors declare no conflicts of interest.

## Supporting information




**Supporting File 1**: advs75430‐sup‐0001‐SuppMat.docx.

**Supporting File 2**: advs75430‐sup‐0002‐DataFile.zip.

## Data Availability

The data that support the findings of this study are available from the corresponding author upon reasonable request.
