# Supplementary material for: Climate Change Threatens Micronutrient Density of European Winter Wheat
Source: Adv Sci (Weinh). 2026 Apr 27;13(42):e13322. doi: 10.1002/advs.202513322 (PMC13335512; doi:10.1002/advs.202513322)

Supporting Information

**Title**

Climate Change Threatens Nutritional Quality of European Winter Wheat

**Authors**

*Da Cao†, Jennifer Michel†, Eline Lorer, Markus Weinmann, Jacques Le Gouis, Claire Léon, Sibille Perrochon, David Alvarez, Vincent Leemans, Iñaki Balanzategui Guijarro, Jordi Moya Laraño, Sara Sánchez Moreno, Symanczik Sarah, Waibel Matthias, Hervé Vanderschuren, Cécile Thonar, Pierre Delaplace, Dominique Van Der Straeten**

†These authors contributed equally.

*Corresponding author: Dominique Van Der Straeten

Email: [Dominique.VanDerStraeten@UGent.be](mailto:Dominique.VanDerStraeten@UGent.be)


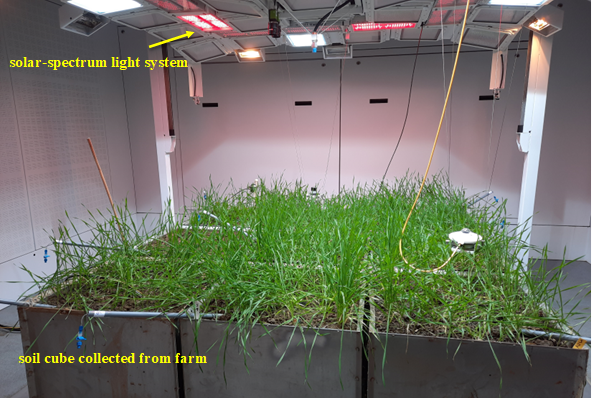


**Figure S1.** The Ecotron facility used in this study. For a detailed description, see [26].


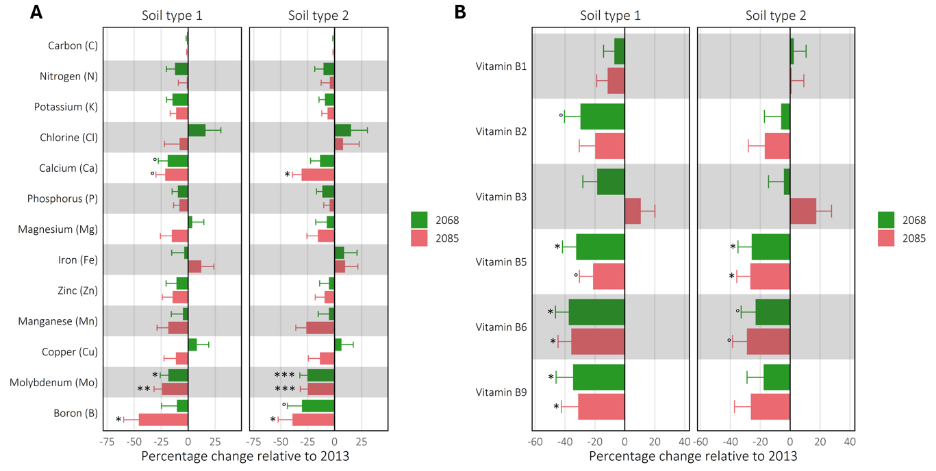
**Figure S2.** Relative carbon, nitrogen, and mineral (**A**) as well as B vitamin (**B**) concentration changes in wheat grains harvested under future meteorological conditions (2068 and 2085) compared to 2013 as the control, under two soil conditions. Values are mean± SE, n=8. Asterisks indicate significant differences in comparisons between future conditions and the year 2013 as the control (*P < 0.05; **P < 0.01; ***P < 0.001, linear mixed model). Specifically, for soil type 1, P = 0.018 and 0.005 for Mo in 2068 and 2085, respectively; P = 0.074 for vitamin B2 and 0.049 for vitamin B5 in 2068; P=0.018 for vitamin B6 and 0.039 for vitamin B9 both in 2068 and 2085. For soil type 2, P = 0.029 for Ca and 0.026 for B in 2085, P =0.00099 for Mo both in 2068 and 2085; P = 0.049 for vitamin B5 both in 2068 and 2085. Circles indicate marginally significant differences, with adjusted P values ranging from 0.1 to 0.05.


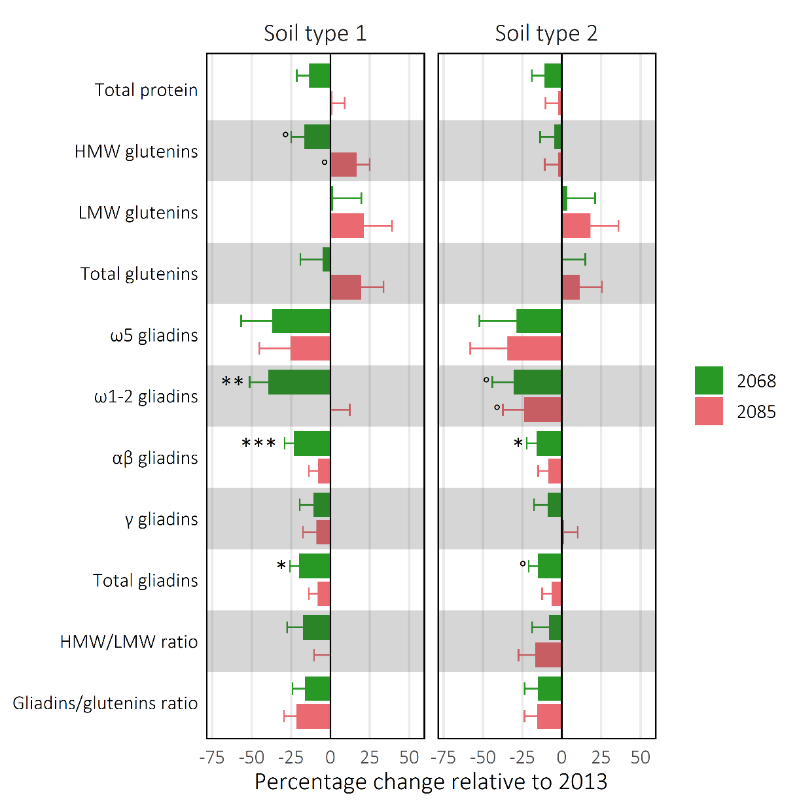


**Figure S3.** Relative protein fraction concentration changes in wheat grains harvested under future meteorological conditions (2068 and 2085) compared to 2013 as the control under two soil conditions Values are mean± SE, n=8. Asterisks indicate significant differences in comparisons between future conditions and the year 2013 as the control (*P < 0.05; **P < 0.01; ***P < 0.001, linear mixed model). Specifically, P = 0.0037 for ω1-2 gladins in 2068 for soil type 1; P = 0.0005 and 0.031 for αβ gliadins in 2068 for soil type 1 and 2 separately; P=0.015 for total gliadins in 2068 for soil type 1. Circles indicate marginally significant differences, with adjusted P values ranging from 0.1 to 0.05.


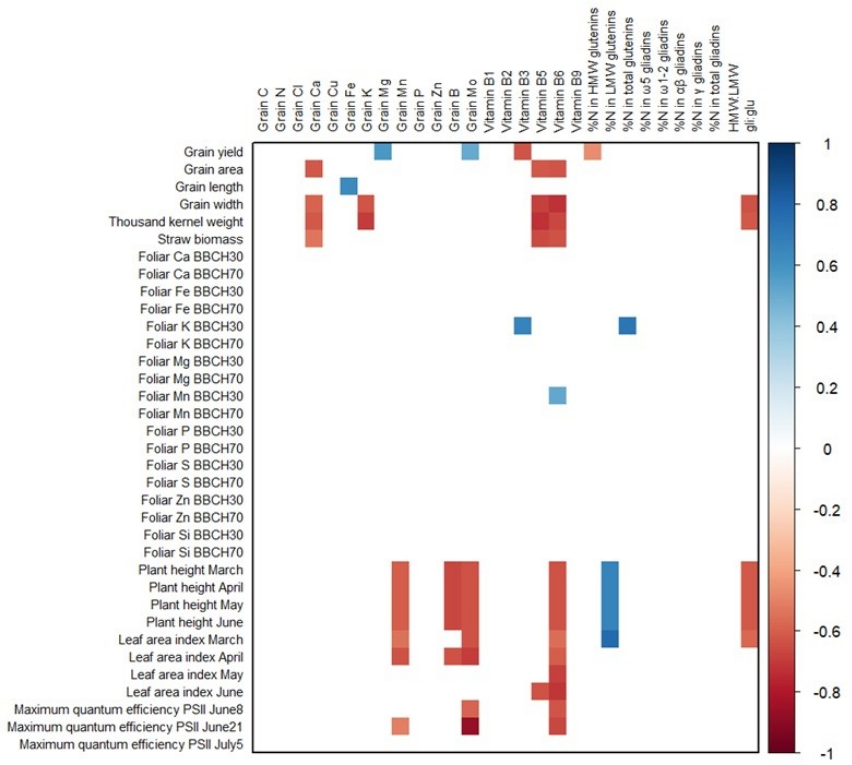


**Figure S4.** Correlation analysis of pairwise Spearman correlations between various plant phenological, physiological, and yield traits and grain quality. Only correlations for FDR adjusted p<0.05 are shown; HMW, high molecular weight; LMW, low molecular weight.

**Table S1.** Absolute element concentration (mean ± SD) in wheat grains harvested under three different meteorological conditions, without (**A**) and with (**B**) splitting across two soil types. S1 represents soil type 1, and S2 represents soil type 2 (for more details on the soil types, see Michel, et al. [26].

**A**

| mg/kg except for C, N | 2013 | | 2068 | | 2085 | |
| --- | --- | --- | --- | --- | --- | --- |
|  | mean | SD | mean | SD | mean | SD |
| Carbon% (C) | 40.6 | 0.9 | 40.2 | 0.2 | 40.3 | 0.2 |
| Nitrogen% (N) | 2.2 | 0.1 | 2.0 | 0.1 | 2.2 | 0.3 |
| Potassium (K) | 5160.5 | 335.6 | 4556.4 | 394.2 | 4704.2 | 338.1 |
| Chlorine (Cl) | 630.6 | 100.5 | 729.8 | 140.5 | 628.5 | 78.9 |
| Calcium (Ca) | 326.0 | 53.5 | 273.1 | 44.4 | 241.6 | 33.6 |
| Phosphorus (P) | 3142.9 | 214.6 | 2814.2 | 226.1 | 2942.6 | 252.8 |
| Magnesium (Mg) | 730.8 | 69.1 | 717.7 | 105.0 | 619.3 | 73.4 |
| Iron (Fe) | 25.8 | 2.4 | 26.4 | 4.1 | 28.6 | 4.1 |
| Zinc (Zn) | 18.2 | 1.7 | 16.8 | 3.2 | 16.1 | 2.5 |
| Manganese (Mn) | 18.2 | 1.7 | 17.3 | 2.8 | 14.2 | 2.2 |
| Copper (Cu) | 2.3 | 0.2 | 2.5 | 0.3 | 2.0 | 0.4 |
| Molybdenum (Mo) | 1.0 | 0.2 | 0.7 | 0.1 | 0.7 | 0.1 |
| Boron (B) | 0.7 | 0.1 | 0.5 | 0.2 | 0.4 | 0.1 |

**B**


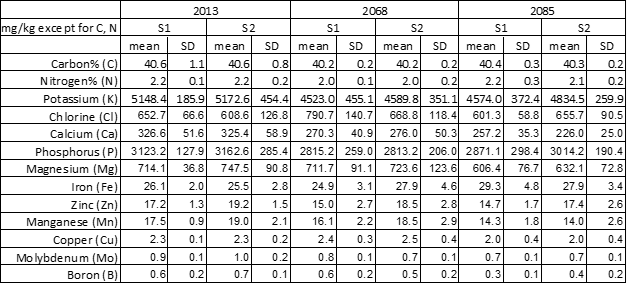


Table S2. Absolute B vitamin concentration (mean ± SD) in wheat grains harvested under three different meteorological conditions, without (a) and with (b) splitting across two soil types. S1 represents soil type 1, and S2 represents soil type 2 (for more details on the soil types, see Michel, et al. [26].

**A**

| µg/100g | 2013 | | 2068 | | 2085 | |
| --- | --- | --- | --- | --- | --- | --- |
|  | mean | SD | mean | SD | mean | SD |
| vitamin B1 (thiamine) | 184.9 | 27.6 | 175.8 | 27.3 | 176.6 | 24.4 |
| vitamin B2 (riboflavin) | 69.6 | 15.2 | 57.0 | 12.6 | 56.8 | 6.1 |
| nicotinic acid | 423.8 | 68.6 | 385.1 | 37.7 | 505.9 | 60.7 |
| nicotinamide | 47.5 | 17.4 | 34.9 | 7.8 | 33.7 | 10.2 |
| vitamin B3 (niacin) | 471.2 | 76.6 | 420.0 | 42.2 | 539.6 | 57.4 |
| vitamin B5 (Pantothenic acid) | 563.1 | 77.3 | 400.4 | 58.5 | 426.6 | 46.7 |
| vitamin B6 (pyridoxine) | 217.8 | 32.4 | 151.3 | 17.1 | 144.9 | 13.3 |
| 5-CHOTHF | 27.0 | 5.8 | 21.0 | 5.1 | 19.8 | 3.2 |
| 5-MeTHF | 6.7 | 1.6 | 4.3 | 1.5 | 4.3 | 1.1 |
| vitamin B9 (folate) | 33.8 | 6.9 | 25.4 | 5.7 | 24.0 | 3.7 |

**B**


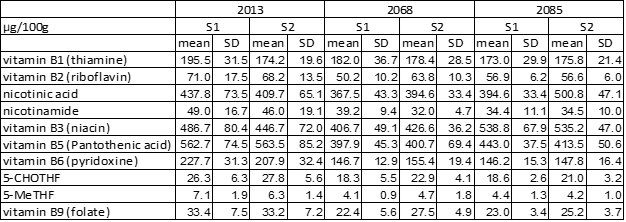

Supplement: Supplementary file 1 — Supporting File 1: advs75430‐sup‐0001‐SuppMat.docx. [file ADVS-13-e13322-s001.docx]
